# Effects of 12 Months Continuous Positive Airway Pressure on Sympathetic Activity Related Brainstem Function and Structure in Obstructive Sleep Apnea

**DOI:** 10.3389/fnins.2016.00090

**Published:** 2016-03-10

**Authors:** Luke A. Henderson, Rania H. Fatouleh, Linda C. Lundblad, David K. McKenzie, Vaughan G. Macefield

**Affiliations:** ^1^Neural Imaging Laboratory, Discipline of Anatomy and Histology, The University of SydneySydney, NSW, Australia; ^2^School of Medicine, Western Sydney UniversitySydney, NSW, Australia; ^3^Department of Respiratory Medicine, Prince of Wales Private HospitalSydney, NSW, Australia; ^4^Neuroscience Research AustraliaSydney, NSW, Australia

**Keywords:** medullary raphe, dorsolateral pons, sleep disordered breathing, hypertension, rostral ventrolateral medulla

## Abstract

Muscle sympathetic nerve activity (MSNA) is greatly elevated in patients with obstructive sleep apnea (OSA) during normoxic daytime wakefulness. Increased MSNA is a precursor to hypertension and elevated cardiovascular morbidity and mortality. However, the mechanisms underlying the high MSNA in OSA are not well understood. In this study we used concurrent microneurography and magnetic resonance imaging to explore MSNA-related brainstem activity changes and anatomical changes in 15 control and 15 OSA subjects before and after 6 and 12 months of continuous positive airway pressure (CPAP) treatment. We found that following 6 and 12 months of CPAP treatment, resting MSNA levels were significantly reduced in individuals with OSA. Furthermore, this MSNA reduction was associated with restoration of MSNA-related brainstem activity and structural changes in the medullary raphe, rostral ventrolateral medulla, dorsolateral pons, and ventral midbrain. This restoration occurred after 6 months of CPAP treatment and was maintained following 12 months CPAP. These findings show that continual CPAP treatment is an effective long-term treatment for elevated MSNA likely due to its effects on restoring brainstem structure and function.

## Introduction

Obstructive sleep apnea (OSA) is characterized by repetitive complete or partial cessation of airflow during sleep, owing to collapse of the upper airways. In addition, it has been shown by numerous investigators that OSA is associated with significantly elevated muscle sympathetic nerve activity (MSNA) during normoxic daytime wakefulness which leads to neurogenic hypertension (Carlson et al., 1993; Narkiewicz et al., 1998; Lanfranchi and Somers, 2001; Narkiewicz and Somers, 2003). Despite the significant effects of increased MSNA on an individual's health, the underlying mechanisms responsible for the increased MSNA in OSA remain unknown.

Over the past decade, numerous investigations have begun to explore the anatomy and function of the brain in individuals with OSA. These studies have established that individuals with OSA display significant gray matter volumetric changes, primarily gray matter concentration reductions in numerous higher brain regions, including some that can significantly modulate changes in MSNA (Macey et al., 2002; Morrell et al., 2003; Canessa et al., 2011). Furthermore, some of these studies have shown that OSA is associated with altered arterial pressure and heart rate responses during numerous challenges and these altered responses appear to be associated with alterations in regional brain activity (Harper et al., 2003, 2012; Henderson et al., 2003; Macey et al., 2003, 2006). Although these studies investigated *evoked* changes in brain activity during various cardiovascular challenges, they did not explore brain sites responsible for the increased *resting* MSNA and associated hypertension in individuals with OSA.

In a series of recent studies we have begun to describe alterations in the neural mechanisms underlying increased resting MSNA in OSA subjects. Using concurrent recording of MSNA and functional magnetic resonance imaging (fMRI) of the brainstem, we found that during each MSNA burst, OSA subjects displayed significantly lower signal intensity changes in the region of the medullary raphe, dorsolateral pons (dlPons), rostral ventrolateral medulla (RVLM), and ventral midbrain (Lundblad et al., 2014). Remarkably we found that these regions also displayed *increased* gray matter concentration in OSA subjects which was significantly correlated to their elevated resting MSNA. Furthermore, in a subsequent investigation we followed these OSA subjects during 6 months of continuous positive airway pressure (CPAP) treatment and found that along with a significant reduction in resting MSNA, the functional and anatomical changes within these brainstem sites returned to control levels (Lundblad et al., 2015).

The aim of this investigation was to extend this latter study by investigating whether the functional and anatomical “restoration” that occurs in OSA subjects after 6 months of CPAP is maintained during an additional 6 months of CPAP treatment, or whether the effects seen at 6 months CPAP are transient in nature. This is important if we are to suggest that continual CPAP treatment is effective at reducing resting MSNA and maintaining normal brainstem function. We hypothesized that the changes in brainstem function and structure observed following 6 months CPAP would be maintained at similar levels following an additional 6 months of CPAP treatment.

## Methods

### Subjects

Fifteen subjects with obstructive sleep apnea (13 males, mean ± SEM age 54.0 ± 2.6, range 35–68 years) and 15 healthy controls (12 males, age 50.0 ± 2.9, 35–68 years) were recruited for the study. All OSA subjects were evaluated and diagnosed during an overnight sleep study (polysomnography) at the sleep laboratory of Prince of Wales Hospital. Each OSA subject was continuously monitored for 8 h using 12-channel polysomnography: electroencephalogram (EEG), electrocardiogram (ECG), and submental electromyogram (EMG) recordings were obtained with surface electrodes. In addition, nasal and oral airflow were recorded using a thermistor and chest and abdominal movements were measured by respiratory inductive plethysmography. Oxyhaemoglobin saturation measurements were obtained using finger pulse oximetry and a microphone was placed on the lower neck to record snoring. Additionally, an ultraviolet light sensitive camera was used to record patient movements during sleep. The recorded data was analyzed offline and the presence of apneas and hypopneas defined according to the international classification of sleep disorders using Alice (Philips Medical Systems, The Netherlands) and Somonologica (Medcare Flaga, Reykjavik, Iceland) software. Based on the full night of respiratory monitoring, the sleep specialist determined the CPAP pressure which was necessary to prevent apnoeic events and subjects were subsequently treated at home with an individually calibrated CPAP machine (Series 9, ResMed, Sydney, Australia). CPAP compliance was based on an automated download of the CPAP machine at 6 and 12 months. Control subjects undertook an overnight assessment using an in-home device that monitored nasal airflow and oxygen saturation (ApneaLink™; ResMed, Sydney, Australia). All procedures were approved by the Human Research Ethics Committees of Western Sydney University and the University of New South Wales. Written consent was obtained from all subjects in accordance with the Declaration of Helsinki. All subjects were included in a series of previous investigations (Fatouleh et al., 2014, 2015; Lundblad et al., 2014, 2015).

### MRI and MSNA acquisition

In the laboratory, subjects lay supine on a MRI bed with their knees supported on a foam cushion. An insulated tungsten microelectrode was inserted precutaneously into a muscle fascicle of the common peroneal nerve to record multiunit MSNA. An uninsulated microelectrode was inserted nearby subdermally (1–2 cm) to act as a reference electrode. Neural activity was amplified (gain 100, band pass 0.1–5.0 kHz) using a MR compatible stainless steel isolated headstage (NeuroAmp Ex. ADInstrument, Australia) and further amplified and filtered (total gain 2 × 10^4^, band pass 0.3–5.0 kHz). Ag-AgCl surface electrodes were used to record ECG (0.3-1.0 kHz), radial arterial tonometry (Colin 7000 NIBP, Colin Corp., Aichi, Japan) used to record continuous non-invasive blood pressure (BP) using and piezoelectric transducer around the abdomen (Pneumotrace, UFI) to record respiration. During a 10 min period of undisturbed rest, spontaneous MSNA, heart rate, respiration, and BP were recorded continuously and the final 5 min used for analysis. Following this recording period, the ECG and BP recordings were stopped and with the microelectrode *in situ*, the subject was placed into the MRI scanner. During the subsequent scanning period, heart rate was monitored using an MR-compatible piezoelectric pulse transducer placed on the fingerpad and respiration was monitored using MR-compatible piezoelectric transducer placed around the abdomen. All physiology data were recorded using a computer-based data acquisition and analysis system (PowerLab 16S; ADInstruments, Australia).

With each subject relaxed and enclosed in a 32-channel SENSE head coil, a continuous series of 200 gradient echo echo-planar image volumes, each volume covering the entire brainstem from the first cervical level to the hypothalamus and sensitive to Blood Oxygen Level Dependent contrast were collected (46 axial slices, TR = 8 s, TE = 40 ms, flip angle 90°, raw voxel size = 1.5 mm^3^) using a 3 Tesla MRI whole body scanner (Achieva, Phillips Medical Systems). A 4 s-ON, 4 s-OFF protocol was used, with MSNA measured during the initial 4 s-OFF period and all 46 axial images in a single brainstem volume collected during the subsequent 4 s-ON period. This was repeated a further 199 times so that a total of 200 periods of MSNA interleaved with 200 periods of brainstem fMRI were collected in each subject. Following this initial scanning session, all 15 OSA subjects began CPAP treatment as described above and returned for further MRI scanning sessions 6 and 12 months later. During these subsequent scanning sessions, T1-weighted anatomical images were collected from all 15 OSA subjects. Concurrent MSNA and brainstem fMRI was collected in 11 of these 15 OSA subjects following both 6 and 12 months of CPAP treatment.

### MSNA and fMRI processing

All MSNA signals were RMS-processed (moving average, time constant 200 ms) and MSNA levels during the pre-MRI recording period were quantified using time-domain analysis of the RMS-processed signal. MSNA levels were quantified as both burst frequency (bursts/min) and burst incidence (bursts/100 heart beats). Significance differences in MSNA levels across each group was determined using an analysis of variance, coupled with Tukey's multiple comparisons test (GraphPad Prism for Mac, version 6.0b, USA). Significant differences were determined between controls and OSA subjects prior to CPAP treatment using unpaired two-tailed, two-sample *t*-test, and between OSA subjects prior to and following 6 and 12 months of CPAP treatment, repeated measures ANOVA was performed. All values quoted are the mean ± SEM and *p* < 0.05 was used to indicate significance. During the fMRI scanning period, MSNA bursts were manually measured from the RMS-processed version of the filtered nerve signal during the 4-s inter-scan OFF period. This period was divided into 4 × 1-s intervals, and the total number of MSNA bursts for each 1-s epoch was determined.

### fMRI processing

With the use of SPM12 software (Friston et al., 1995), brainstem fMRI images were realigned and coregistered to each subjects own T1-weighted image set. No subject displayed movement parameters greater than 1 mm in any direction and, as a result, all subjects were used in the final analysis. Furthermore, manual correction was performed to create a precise match with the wholebrain anatomical image set for each subjects. A linear detrending method was then employed to remove non-specific global signal intensity drifts and using the SUIT toolbox (Diedrichsen, 2006), the brainstem and cerebellum were isolated from the wholebrain and the fMRI images spatially normalized into Montreal Neurological Institute (MNI) space using a spatially unbiased atlas template. The fMRI images were then spatially smoothed using a 3 mm full-width half-maximum Gaussian filter.

**Figure 1:**
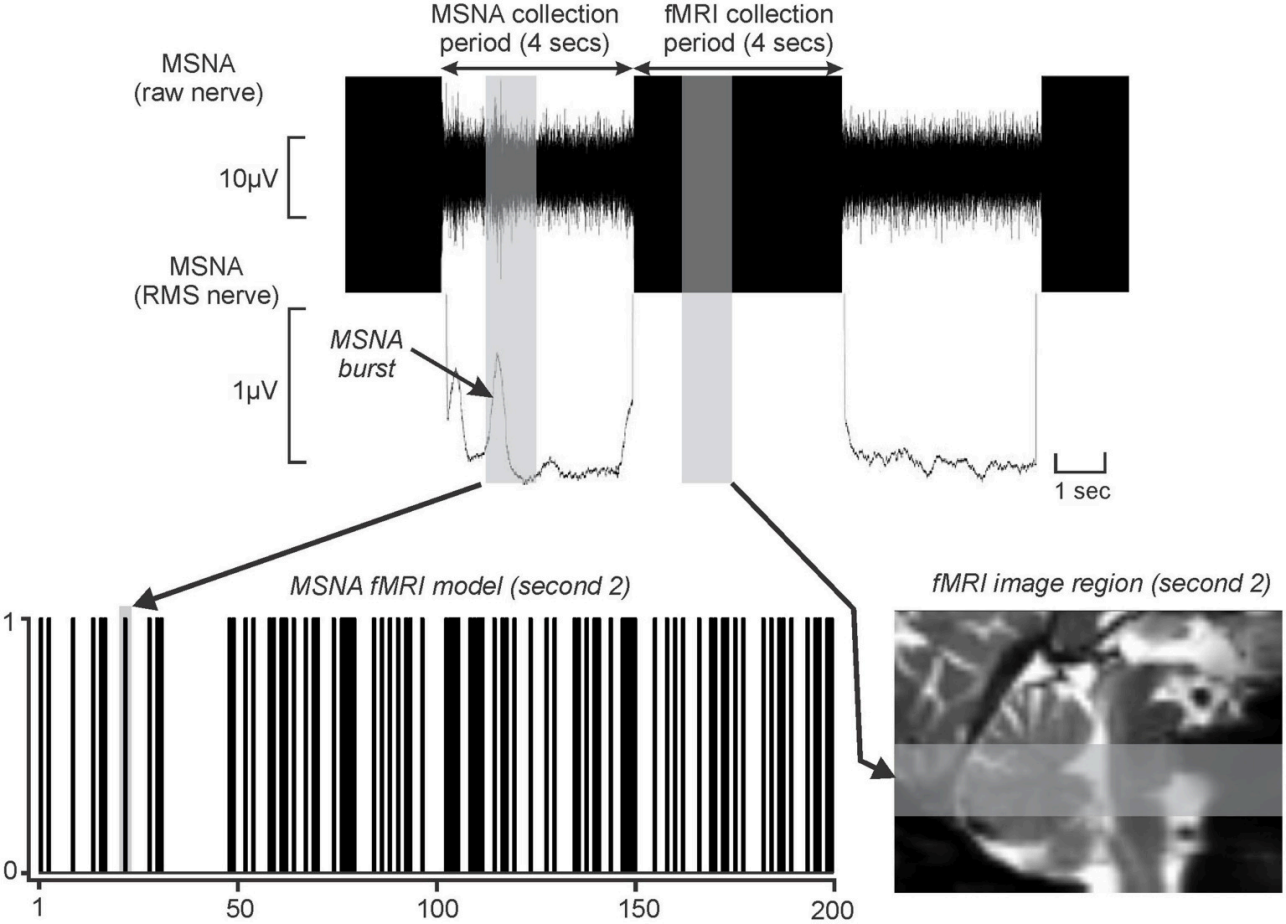
**Upper panel shows a typical microneurographic recording in an individual subject during concurrent functional magnetic resonance imaging (fMRI)**. Brainstem images were collected during a 4 s at which time muscle sympathetic nerve activity (MSNA) activity was not distinguishable. However, due to the fMRI haemodynamic delay (~5 s) and the delay for MSNA traffic to travel from the brain to the recording electrode (~1 s), brain activity during the MSNA collection period was reflected in signal intensity changes during the subsequent 4 s period. The vertical gray shading represents a 1 s period of MSNA recording and the associated 1 s period of fMRI image collection. During each 1 s epoch of the collection period (4 s), if a MSNA burst occurred, a “1” was entered into an fMRI search model (lower left panel). This was repeated for each of the 200 fMRI volumes to create a 200 point fMRI model. The MSNA burst in the above recording is entered into the fMRI search model (vertical gray bar). The MSNA burst that occurred during the 2nd second of the collection period arose during collection of fMRI images through the rostral medulla and pons. This brainstem region is represented by the gray horizontal shading on a sagittal section of an individual's fMRI images set (lower right panel). RMS: root mean square. This figure is modified from our previous publication (Fatouleh et al., 2015).

For each of the 200 fMRI image volumes, the 4 s during which brainstem BOLD signals were recorded were related to the MSNA burst in the preceding 4-s period. This is possible because (1) the delay in neurovascular coupling mean that BOLD signal intensity changes lag neuronal events by ~5 s (Logothetis et al., 2001), and (2) slow conduction speeds of unmyelinated peripheral axons means it requires ~1 s for a sympathetic burst to be transmitted from the brain to the peripheral recording site (Fagius and Wallin, 1980); as a consequence of these two factors, it is apparent that (3) a BOLD signal intensity change would occur ~4 s after an increase in neuronal activity within the brain. Furthermore, since we collected brain images in a caudal-to-rostral direction, we could target specific brainstem regions given the timing relationship between scanning acquisition and structure (Figure 1). Thus, for each subject, the brainstem was divided into four sections from caudal to rostral corresponding to the first, second, third, and fourth 1-s periods and a brain mask was created for each of these 1 s periods. The fourth 1-s period was discarded as it covered the region rostral to the brainstem. The MSNA recordings were also then divided into corresponding 1-s periods.

For each 1-s epoch, a “1” was entered into an fMRI search model if a MSNA burst occurred and a “0” if no burst occurred. This process was repeated resulting in a 200-volume fMRI search model for each of the 3 × 1-s periods. Figure 1 shows an example of a MSNA burst model created using this procedure. A general linear model approach was then used to determine changes in BOLD signal intensity that matched each individual subject's MSNA burst model for each of the 1-s epochs. Since we had accounted for the hemodynamic delay in our methodological setup, the hemodynamic delay function and microtime resolution were removed from the analysis. Additionally, for each subject, their six directional movement parameters derived from the realignment step were added as nuisance variables. We also included signal changes derived from a 2-mm sphere placed in the center of the fourth ventricle as a nuisance variable, in an attempt to eliminate the effects of heart rate.

We used second level analyses to compare signal intensity changes during each MSNA burst in OSA subjects prior to CPAP and following 6 and 12 months of CPAP treatment. Any significant difference in MSNA-related signal intensity change in each individual subject over the three time points (pre-CPAP, 6 months CPAP, 12 months CPAP) were determined using an F test and the results displayed using a threshold of *p* < 0.005 uncorrected for multiple comparisons. During this second-level analysis, the resulting statistical maps were masked with the brain or brainstem region corresponding to the relevant 1-s epochs. We have previously explored brainstem functional and anatomical changes in 13 OSA subjects following 6 months CPAP treatment (Lundblad et al., 2014, 2015) and 11 of these OSA subjects returned following 12 months CPAP treatment for this investigation. Furthermore, the 15 OSA and 15 control subjects in which T1-weighted anatomical images were used to explore changes in gray matter concentration were also used in previous investigations (Fatouleh et al., 2014, 2015; Lundblad et al., 2014, 2015). Given that this study is essentially an extension of this earlier investigation and we hypothesized that CPAP treatment would result in signal change restoration and maintenance in regions we previously showed to change following 6 months CPAP, we employed small volume correction for multiple comparisons (*p* < 0.05).

It is possible that differences in activation patterns were partially due to differences in the number of MSNA bursts in controls compared to OSA subjects since we were correlating ongoing signal intensity fluctuations with spontaneous MSNA fluctuations. That is, differences in the number of ON and OFF periods could influence the overall significance of the final contrast maps, which in turn may have influenced the second-level analyses. To reduce this possibility, for each significant group of three or more contiguous voxels that survived the statistical threshold (significant cluster), we extracted the raw signal intensity changes in OSA subjects before and after CPAP treatment, as well as in control subjects, and compared signal intensity during bursts of MSNA to signal intensity during periods where there were no bursts. For each significant cluster, the mean (±SEM) signal intensity changes for each group were plotted. Significant differences in signal intensity between controls and OSA subjects (two-sample *t*-test, *p* < 0.05) and significant differences in OSA subjects before and following 6 and 12 months CPAP treatment were determined (two-tailed, paired *t*-test, *p* < 0.05). Finally, for each significantly different cluster, linear relationships between AHI-values and the difference in fMRI signal intensity change pre-CPAP compared with the mean of the control subjects and in OSA subjects pre-CPAP compared with 6 and 12 months post-CPAP were determined (*p* < 0.05).

### T1 image processing

Using the SUIT toolbox, the wholebrain images were cropped and the brainstem masked to remove all supratentorial gray matter. The brainstem images were then spatially normalized with a dedicated symmetrical brainstem template, spatially normalized into the brainstem template space and modulated by the volume changes due to the normalization. Finally, the resulting “maps” of gray matter probabilities were smoothed using a 3 mm full-width half-maximum Gaussian filter. Second-level random-effects analyses were performed to compare gray matter volume (concentration) changes in OSA subjects prior to CPAP and following 6 and 12 months of CPAP treatment. Any significant change in gray matter volume in each individual subject over the three time points (pre-CPAP, 6 months CPAP, 12 months CPAP) were determined using an F test and the results displayed using a threshold of *p* < 0.05 false discovery rate corrected for multiple comparisons. For each significant cluster, gray matter volumes were extracted in OSA subject's pre-CPAP, 6 months CPAP and 12 months CPAP as well as from controls and the mean (±SEM) plotted. Significant differences in gray matter volume between controls and OSA subjects (two-sample *t*-test, *p* < 0.05) were determined. Finally, for each significantly different cluster, linear relationships between AHI-values and the difference in gray matter volume pre-CPAP compared with the mean of the control subjects and in OSA subject's pre-CPAP compared with 6 and 12 months post-CPAP were determined (*p* < 0.05).

## Results

### OSA subject characteristics

Although, all OSA and control subjects were included in a series of previous investigations and thus some of the diagnostic results have been published previously, we have included them in this manuscript for completeness. OSA subjects were diagnosed on the basis of their apnea-hypopnea index (AHI)-values (apnea-hypopnea events per hour) as mild, moderate, or severe (mild: AHI 5–15; moderate: AHI 15–30; severe: AHI > 30). Of the 15 OSA subjects, 2 subjects had mild OSA, 1 subject had moderate OSA, and 12 subjects had severe OSA (AHI 37.2 ± 4.1; range 7–62). The minimum SaO_2_ during sleep was 82.7 ± 2.0% (range 67–93%); the baseline SaO_2_ during wakefulness was 94.9 ± 0.7% (range 91–99%), and the Baseline Epworth Sleep Scale score was 8.6 ± 1.2 (range 3–19). We found that OSA patients used CPAP for an average of 5.5 ± 0.3 h/night during the first 6 month period and 5.5 ± 0.3 h/night during the 6–12 month period and there was a significant reduction in AHI after 6 months of CPAP treatment (AHI 3.0 ± 0.8; range 0.6–9.9) which reduced further after 12 months treatment (AHI 1.6 ± 0.4; range 0.3–7.1). Although, the mean AHI for control subjects was only 3.0 ± 1.0, we did find that two of the control subjects had an AHI of 8 and 10. We did not exclude these subjects since they were asymptomatic normotensive and did not report being tired during the day or snoring during sleep; we did not consider it necessary to undertake a full polysomnographic assessment in these two subjects. There was no difference in age between OSA and control groups (two-sample *t*-test; *p* > 0.05) and although OSA subjects had greater body mass index than controls during the first MRI session (BMI: pre-CPAP 31.3 ± 1.5, controls 25.1 ± 1.1; *p* = 0.0014), their BMI did not alter between MRI scanning sessions [pre-CPAP: 31.3 ± 1.5; 6 months CPAP 31.5 ± 1.9; 12 months CPAP: 31.0 ± 3.1; *p* > 0.05; *F*_(2, 30)_ = 0.0159; *p* = 0.9842].

### Physiology

**Figure 2:**
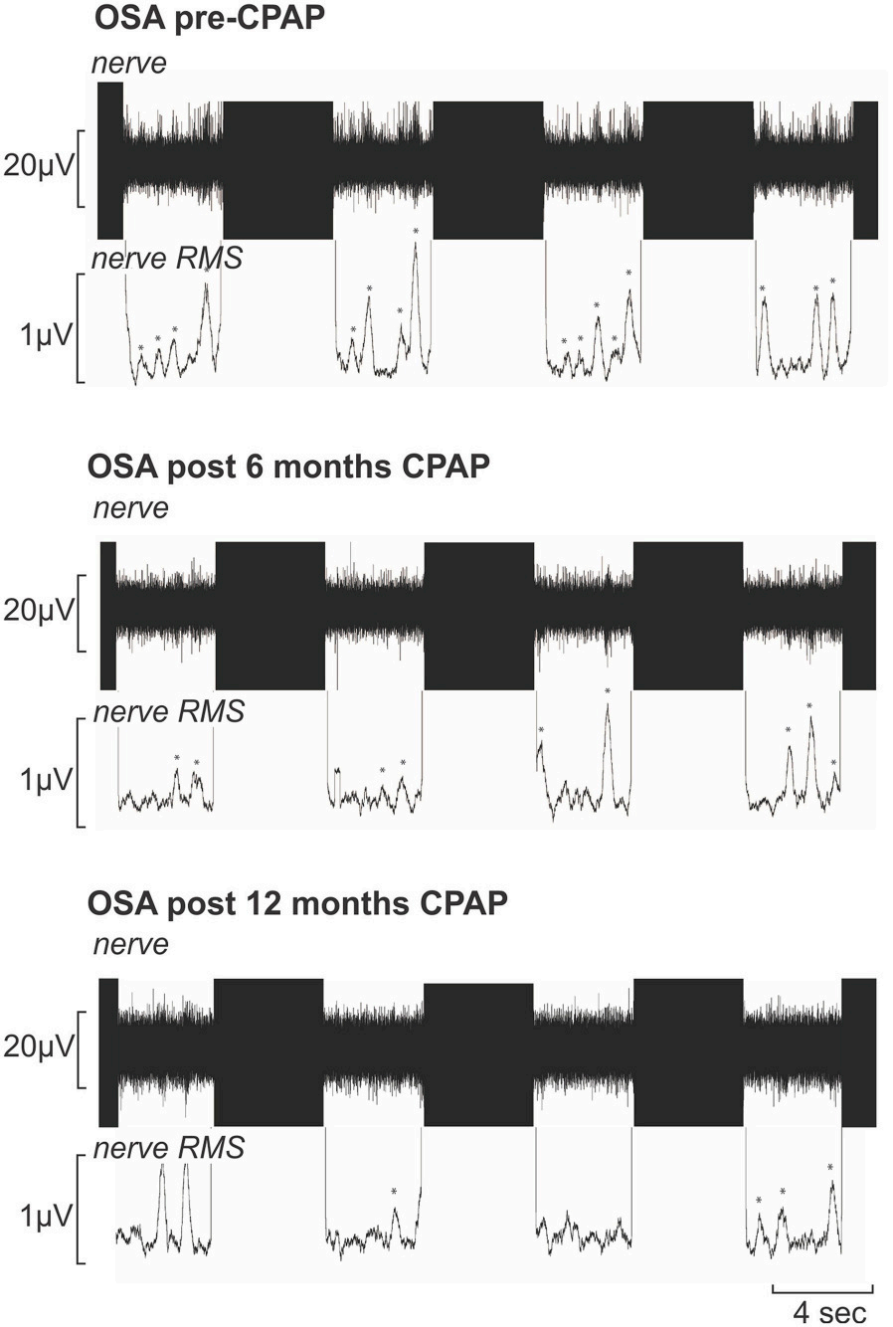
**Multiunit recording of muscle sympathetic nerve activity (MSNA) from a 50-year-old male patient with obstructive sleep apnea (OSA) acquired during functional magnetic resonance imaging sessions prior to, following 6 months and following 12 months of continuous positive airway pressure (CPAP) treatment**. Each recording shows four consecutive sets of the 4s-ON and 4s-OFF scanning sequences; the black areas represent the scanning artifacts. MSNA burst amplitudes were measured during the OFF periods. The mean-voltage neurogram is shown in the nerve RMS (root mean square) trace; this was used to quantify the number of sympathetic bursts. Note the high number of MSNA bursts (identified by ^*^) pre-CPAP which is reduced following 6 and 12 months of CPAP treatment.

**Figure 3:**
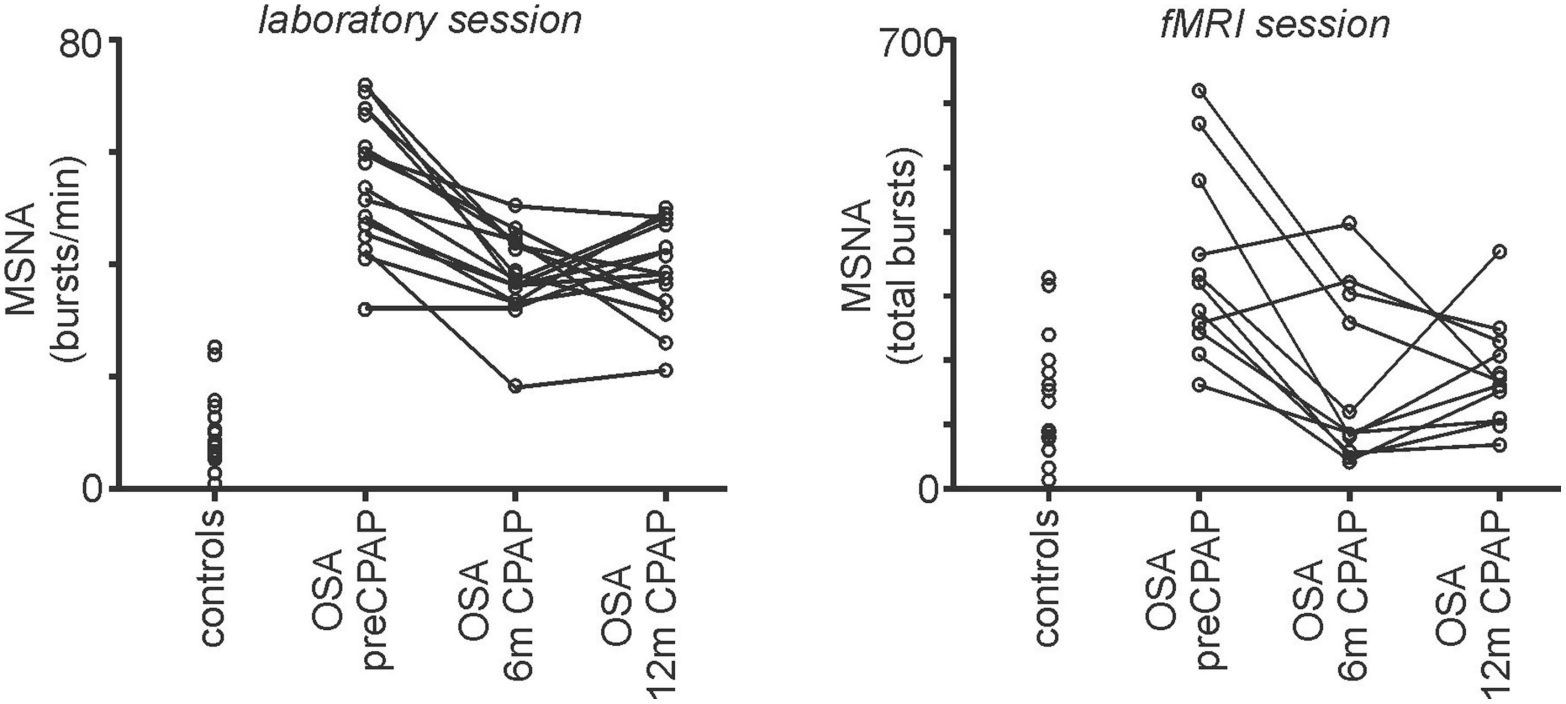
**Plots of muscle sympathetic nerve activity (MSNA) during the laboratory session and the brainstem functional magnetic resonance imaging (fMRI) session in controls and in individuals with obstructive sleep apnea (OSA) prior to and following 6 and 12 months of continuous positive airway pressure (CPAP) treatment**. Individual values of bursts/min for the laboratory session and total MSNA bursts during the fMRI session are represented by the open circles. Note that CPAP results in a reduction in resting MSNA toward control levels.

During the laboratory recording session, compared with controls, OSA subjects (*n* = 15) had significantly elevated systolic (controls vs. OSA: 121.4 ± 4.0 vs. 137.7 ± 4.9 mmHg; *p* = 0.04) and diastolic (67.9 ± 3.6 vs. 80.6 ± 2.1 mmHg; *p* = 0.004) pressures before CPAP. Furthermore, MSNA burst frequency (24.2 ± 2.4 vs. 54.2 ± 3.1 bursts/min; *p* < 0.0001) and burst incidence (37.7 ± 3.2 vs. 79.0 ± 5.7 bursts/100 heart beats; *p* < 0.0001) were both significantly elevated in OSA subjects compared with controls before CPAP. Repeated-measures analysis of variance revealed significant change in burst frequency and burst incident at 0 (baseline), 6 and 12 months of compliant CPAP use. Six months of CPAP treatment resulted in on average, a significant reduction in resting MSNA which was maintained at 12 months CPAP treatment [*burst frequency:* pre-CPAP: 54.2 ± 3.1; 6 months CPAP: 37.9 ± 2.0; 12 months CPAP: 38.0 ± 2.4; *F*_(1.552, 21.73)_ = 18.14; *p* < 0.0001; *burst incidence:* pre-CPAP: 79.0 ± 5.7; 6 months CPAP: 56.9 ± 3.2; 12 months CPAP: 49.4 ± 2.2; *F*_(2, 38)_ = 16.55; *p* < 0.0001]. Figure 2 shows physiology recordings from one OSA subject pre-CPAP, following 6 months and 12 months of CPAP treatment during their three fMRI scanning sessions. It is clear that CPAP results in a reduction in the number of MSNA bursts during each fMRI scanning period. Consistent with the lab sessions, the reduction in MSNA resulting from CPAP treatment was also evident during the fMRI sessions in the 11 OSA subjects that completed this part of the experiment (*total bursts during brainstem fMRI scan:* controls: 151 ± 24; pre-CPAP: 350 ± 37; 6 months CPAP: 167 ± 33; 12 months CPAP: 182 ± 21; Figure 3).

### Brainstem fMRI signal intensity changes

**Figure 4:**
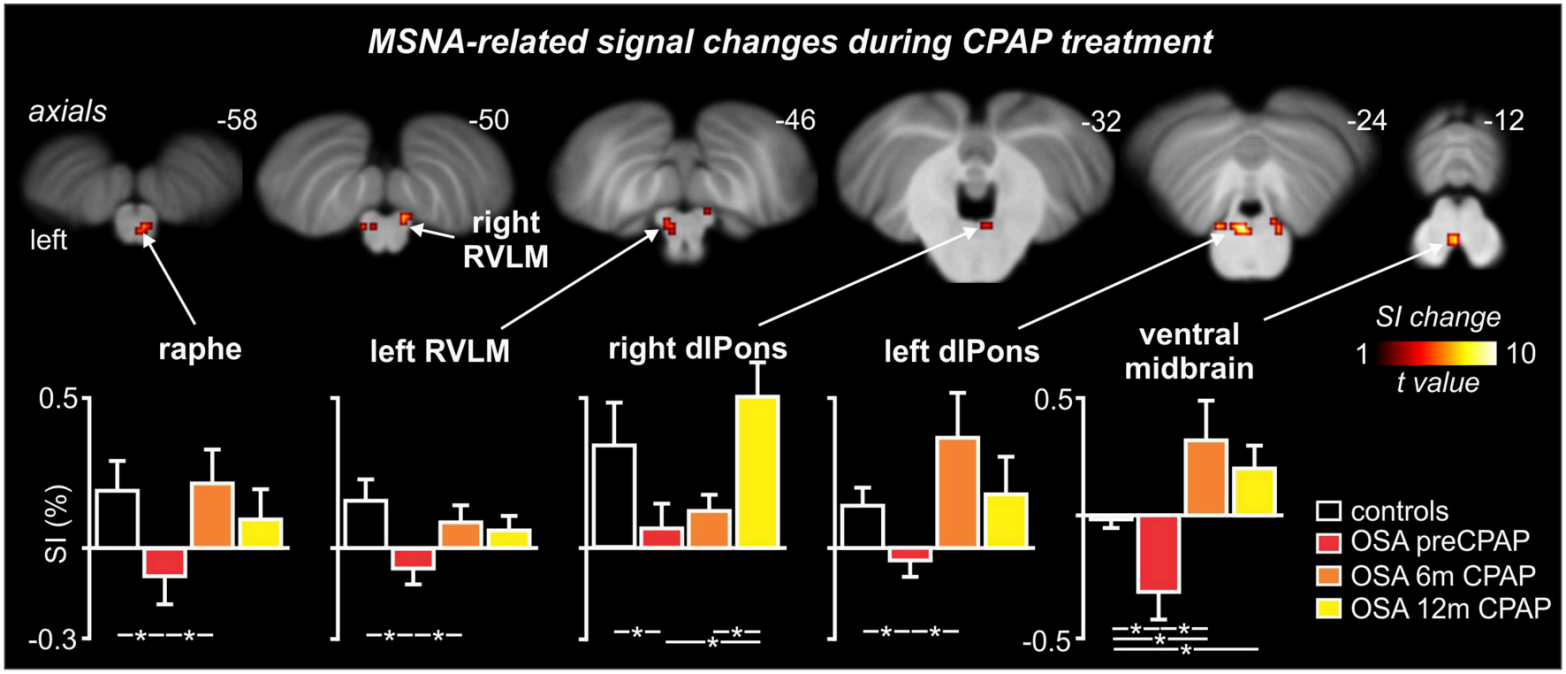
**Brainstem regions in which fMRI signal intensity changes correlated to muscle sympathetic nerve activity (MSNA) were significantly altered by continuous positive airway pressure (CPAP) treatment over 12 months in subjects with obstructive sleep apnea (OSA)**. Hot color scale indicates brainstem regions in which signal intensity (SI) changes were significantly altered by CPAP treatment. Significant clusters are overlaid onto a T1-weighted anatomical template image. Slice location in Montreal Neurological Institute space are indicated at the upper right of each image. The lower panel shows plots of mean (±SEM) percentage changes in signal intensity during each MSNA burst compared to periods of no MSNA bursts for each significant cluster in controls and OSA subjects prior to and following 6 and 12 months of CPAP treatment. Significant difference were determined using two-sample and paired *t*-tests (^*^p < 0.05). dlPons, dorsolateral pons; RVLM, rostral ventrolateral medulla. Note that for most brainstem regions, CPAP treatment restores brainstem function to control levels.

MSNA-related BOLD signal intensity changes pre-CPAP and following 6 and 12 months CPAP revealed significant changes in MSNA-related signal intensity changes in a numerous brainstem regions (Figure 4). Significantly signal intensity changes occurred in the region of the caudal medullary raphe, left and right rostral medulla in the region of the rostral ventrolateral medulla (RVLM), left and right dorsolateral pons (dlPons) in the region of the parabrachial nucleus and in the ventral midbrain in the region of the ventral tegmental area. Comparison of percentage changes in signal intensity during MSNA bursts, compared with periods of no bursts, showed that CPAP restored brainstem activity to control levels in most of these regions. Within the medullary raphe, RVLM, left and right dlPons, MSNA-related signal intensity was reduced pre-CPAP compared to controls but then increased to control levels following CPAP treatment (see Table 1 for mean signal intensity changes). The ventral midbrain was also significantly reduced in OSA subjects pre-CPAP compared with controls and although CPAP treatment reversed this decrease it resulted in an increase that remained above control levels following CPAP treatment. Finally, signal intensity changes pre-CPAP compared with controls or in OSA following 6 and 12 months of CPAP treatment in all of these brainstem regions were not significantly correlated to individual subject's OSA severity (*raphe:* 0 m: *r* = 0.11, *p* = 0.75, 6 m: *r* = 0.02, *p* = 0.96, 12 m: *r* = 0.32, *p* = 0.33; *left RVLM:* 0 m: *r* = 0.19, *p* = 0.58, 6 m: *r* = 0.21, *p* = 0.54, 12 m: *r* = 0.12, *p* = 0.73; *right dlPons:* 0 m: *r* = 0.01, *p* = 0.99, 6 m: *r* = 0.04, *p* = 0.91, 12 m: *r* = 0.18, *p* = 0.61; *left dlPons:* 0 m: *r* = 0.37, *p* = 0.26, 6 m: *r* = 0.20, *p* = 0.55, 12 m: *r* = 0.44, *p* = 0.18; *ventral midbrain:* 0 m: *r* = 0.11, *p* = 0.75, 6 m: *r* = 0.23, *p* = 0.50, 12 m: *r* = 0.19, *p* = 0.58).

**Table 1:** **Locations and mean signal intensity changes in muscle sympathetic nerve (MSNA) related changes in blood oxygen level dependent signal intensity in controls and subjects with obstructive sleep apnea (OSA) prior and following 6 and 12 months of continuous positive airway pressure (CPAP) treatment**.

**Brainstem region**	**MNI co-ordinate**			**Cluster size**	***F*-score**	**Mean (**±***SEM*****) signal intensity change**			
	**x**	**y**	**z**			**Controls**	**OSA pre-CPAP**	**OSA 6 m CPAP**	**OSA 12 m CPAP**
Medullary raphe	2	−40	−58	11	5.90	0.16 ± 0.13	−0.12 ± 0.09^*^	0.22 ± 0.11^†^	0.10 ± 0.10
RVLM									
Left	−4	−36	−44	6	4.14	0.16 ± 0.07	−0.07 ± 0.07^*^	0.06 ± 0.06^†^	0.05 ± 0.06
Right	10	−44	−48	6	7.20	0.12 ± 0.08	−0.08 ± 0.06^*^	−0.18 ± 0.09^*^^†^	0.08 ± 0.07^‡^
dlPons									
Left	−2	−30	−22	51	12.31	0.16 ± 0.06	−0.02 ± 0.09^*^	0.34 ± 0.17^†^	0.18 ± 0.11
Right	4	−38	−32	5	4.08	0.37 ± 0.15	0.08 ± 0.08^*^	0.12 ± 0.06	0.53 ± 0.14^†^^‡^
	8	−34	−24	7	2.90				
Ventral midbrain	0	−22	−14	16	2.78	−0.01 ± 0.05	−0.39 ± 0.14^*^	0.30 ± 0.18^*^^†^	0.19 ± 0.11^*^^†^

*Locations are given in Montreal Neurological Institute space*.

* *Significantly different to controls*.

† *Significantly different to OSA pre-CPAP*.

‡ *Significantly different to OSA 6 months CPAP (p < 0.05)*.

*dlPons, dorsolateral pons; RVLM, rostral ventrolateral medulla*.

### Brainstem gray matter concentration changes

**Figure 5:**
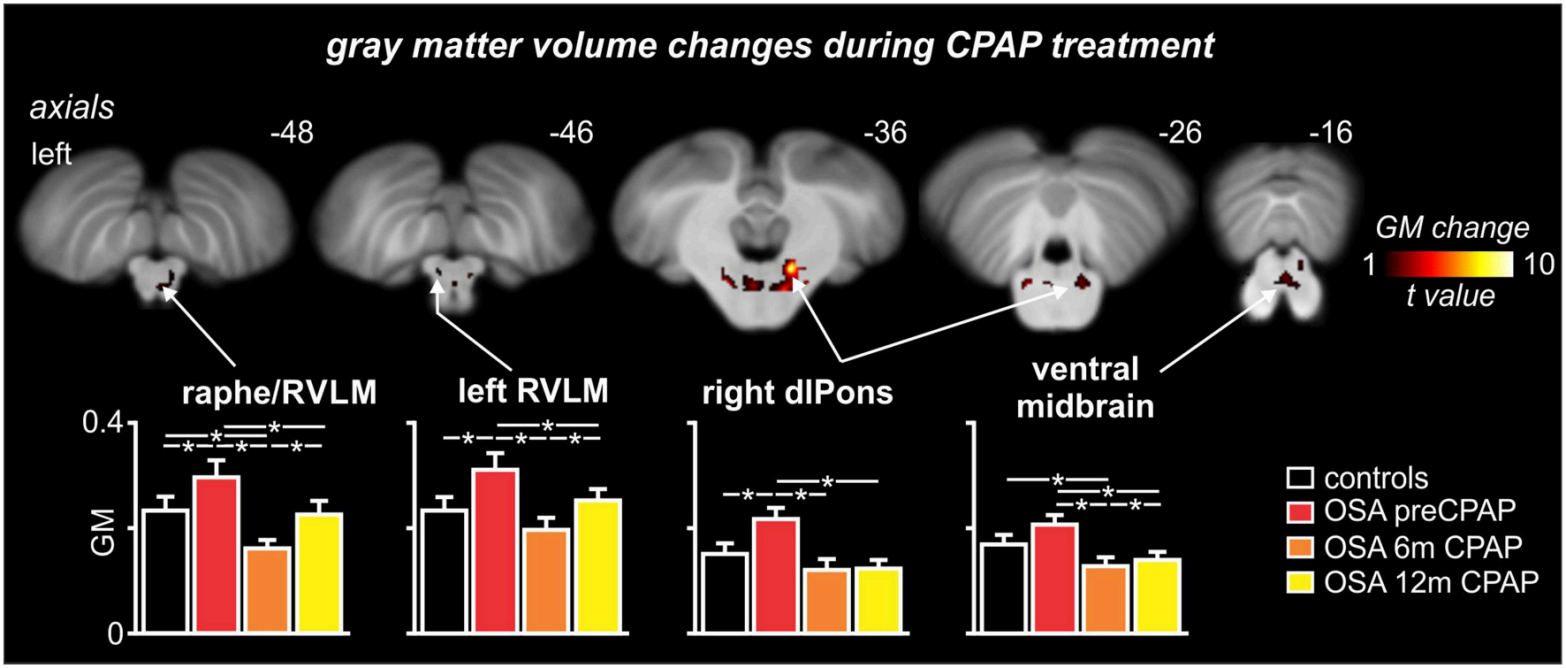
**Brainstem regions in which gray matter (GM) concentrations were significantly altered by continuous positive airway pressure (CPAP) treatment over 12 months in subjects with obstructive sleep apnea (OSA)**. Hot color scale indicates brainstem regions in which gray matter concentration changes were significantly altered by CPAP treatment. Significant clusters are overlaid onto a T1-weighted anatomical template image. Slice location in Montreal Neurological Institute space are indicated at the upper right of each image. The lower panel shows plots of mean (±SEM) gray matter concentrations for each significant cluster in controls and OSA subjects prior to and following 6 and 12 months of CPAP treatment. Significant difference were determined using two-sample and paired *t*-tests (^*^p < 0.05). dlPons, dorsolateral pons; RVLM, rostral ventrolateral medulla. Note that for most brainstem regions, CPAP treatment restores brainstem anatomy to control levels.

Comparison of gray matter concentrations resulted in a remarkably similar set of brainstem structures to those displaying functional differences. Gray matter concentration increases in OSA subjects pre-CPAP compared with controls occurred in the medullary raphe, the region encompassing the RVLM, the dlPons and in the ventral midbrain (Figure 5). As with the functional changes, within all of these regions, gray matter concentration was returned to levels not different to controls following 12 months of CPAP treatment (see Table 2 for gray matter concentrations). Within the medullary raphe and ventral midbrain, gray matter concentration following 6 months CPAP rebounded to levels marginally below control levels before returning to levels not different to controls following 12 months CPAP. Within the left RVLM and dlPons, gray matter returned to control levels after 6 months CPAP and then remained at control levels following 12 months CPAP. Similar to the fMRI signal changes, gray matter concentration changes pre-CPAP compared with controls or in OSA following 6 and 12 months of CPAP treatment in all of these brainstem regions were not significantly correlated to individual subject's OSA severity (*raphe/RVLM:* 0 m: *r* = 0.10, *p* = 0.71, 6 m: *r* = 0.04, *p* = 0.89, 12 m: *r* = 0.12, *p* = 0.66; *left RVLM:* 0 m: *r* = 0.25, *p* = 0.36, 6 m: *r* = 0.28, *p* = 0.30, 12 m: *r* = 0.31, *p* = 0.26; *right dlPons:* 0 m: *r* = 0.20, *p* = 0.49, 6 m: *r* = 0.23, *p* = 0.40, 12 m: *r* = 0.03, *p* = 0.92; *left dlPons:* 0 m: *r* = 0.09, *p* = 0.76, 6 m: *r* = 0.11, *p* = 0.70, 12 m: *r* = 0.18, *p* = 0.52; *ventral midbrain:* 0 m: *r* = 0.10, *p* = 0.73, 6 m: *r* = 0.06, *p* = 0.83, 12 m: *r* = 0.07, *p* = 0.81).

**Table 2:** **Locations of gray matter concentration differences in controls compared with subjects with obstructive sleep apnea (OSA) prior and following 6 and 12 months of continuous positive airway pressure (CPAP) treatment**.

**Brainstem region**	**MNI co-ordinate**			**Cluster size**	***F*-score**	**Mean (**±**SEM) signal intensity change**			
	**x**	**y**	**z**			**controls**	**OSA pre-CPAP**	**OSA 6 m CPAP**	**OSA 12 m CPAP**
Raphe/Right RVLM	2	−34	−47	187	12.95	0.24 ± 0.02	0.31 ± 0.02^*^	0.16 ± 0.02^*^^†^	0.23 ± 0.02^‡^^‡^
	6	−38	−46						
Left RVLM	−5	−39	−46	10	10.16	0.24 ± 0.02	0.31 ± 0.03^*^	0.20 ± 0.02^†^	0.25 ± 0.02^‡^^‡^
dlPons									
Left	13	−39	−37	852	57.38	0.14 ± 0.02	0.19 ± 0.02^*^	0.09 ± 0.01^*^^†^	0.08 ± 0.01^*^^†^
Right	−10	−32	−33	50	22.15	0.16 ± 0.02	0.22 ± 0.02^*^	0.12 ± 0.01^†^	0.12 ± 0.01^†^
Ventral midbrain	−2	−27	−18	120	26.56	0.17 ± 0.02	0.21 ± 0.02	0.13 ± 0.01^*^^†^	0.14 ± 0.01^†^^‡^

*Locations are given in Montreal Neurological Institute space*.

* *Significantly different to controls*.

† *Significantly different to OSA pre-CPAP*.

‡ *Significantly different to OSA 6 months CPAP (p < 0.05)*.

*dlPons, dorsolateral pons; RVLM, rostral ventrolateral medulla*.

## Discussion

We found that the increase in resting MSNA in individuals with OSA was significantly reduced toward control levels following 6 and 12 months of CPAP treatment. This MSNA reduction was associated with restoration of MSNA-related activity and structural changes in the medullary raphe, RVLM, dlPons and ventral midbrain. This restoration occurred after 6 months of CPAP treatment and was maintained following 12 months CPAP. These findings show that continual CPAP treatment is an effective long-term treatment for elevated MSNA likely due to its effects on restoring brainstem structure and function.

We and others have shown that the elevated MSNA associated with OSA is significantly reduced by CPAP treatment (Hedner et al., 1988; Narkiewicz et al., 1999a; Fatouleh et al., 2015; Lundblad et al., 2015). Interestingly, we found that during the fMRI sessions, during the first 6 months of CPAP, MSNA decreased dramatically and was maintained at these lower levels. Associated with the reduction in MSNA were significant functional changes in a number of brainstem regions including the medullary raphe, RVLM, dlPons, and ventral midbrain. In these regions, signal intensity was significantly reduced in OSA subjects pre-CPAP compared to controls, but returned to or increased above control levels following 6 months CPAP and remained at these levels at the 12 month CPAP scan. It is well-established that the RVLM contains premotor neurons which project directly to the sympathetic preganglionic neurons in the intermediolateral cell column of the spinal cord (Dampney, 1994) and human imaging studies support its role in mediating evoked changes in sympathetic drive and resting sympathetic activity (Macefield et al., 2006; Macefield and Henderson, 2010; Sander et al., 2010). Brainstem sites such as the medullary raphe and dlPons can mediate MSNA changes via their direct projections to the RVLM (Saper and Loewy, 1980; Carrive et al., 1988; Lovick, 1992) although the medullary raphe also projects directly to the intermediolateral cell column (Loewy, 1981). Surprisingly, despite elevated MSNA, OSA subjects displayed decreases in MSNA-related signal intensity within the RVLM which subsequently increased during CPAP treatment. These signal changes appear at odds with the finding that OSA is associated with elevated MSNA and our previous report that RVLM signal intensity increases during each MSNA burst in healthy controls (Macefield and Henderson, 2010). Furthermore, the chronic intermittent hypoxia (CIH) model of OSA which is characterized by chronically elevated sympathetic activity and increased arterial pressure (Fletcher et al., 1992; Bao et al., 1997) is also associated with increased neural activity in the RVLM (Greenberg et al., 1999; Knight et al., 2011). It is possible that the decrease in RVLM signal intensity in OSA subjects reflects altered drive from the raphe, dlPons and/or the ventral midbrain. Indeed, reduced inhibitory drive onto the RVLM from these other brainstem sites would likely result decreased signal intensity but increased RVLM output onto the preganglionic sympathetic neurons in the intermediolateral cell column.

OSA subjects also displayed signal decreases within the medullary raphe, dlPons and ventral midbrain that returned to control levels at 6 months CPAP and remained at control levels at 12 months CPAP. Although in experimental animals CIH does not increase dlPons resting activity, it does result in altered reactivity with enhanced activity during acute stressors (Ma et al., 2008). While inactivation of the either the dlPons or the medullary raphe does not alter resting arterial pressure and heart rate (Henderson et al., 2000; Shafei et al., 2011), chemical stimulation can evoke increases or decreases in arterial pressure, and sympathetic nerve activity (Hade et al., 1988; Miyawaki et al., 1991; Dampney, 1994; Coleman and Dampney, 1995; Henderson et al., 1998). The cardiovascular responses to medullary raphe stimulation appear to be mediated via projections to the RVLM since bilateral micro-injections of bicuculline into RVLM sympathoexcitatory sites abolishes the depressor and sympathoinhibitory response evoked by medullary raphe stimulation (Coleman and Dampney, 1998). Similarly, the dlPons can alter cardiovascular function via the RVLM since stimulation of the parabrachial nucleus in the dlPons increases glutamate release in the RVLM and supresses baroreflex bradycardia (Len and Chan, 1999). A clear role for the ventral midbrain in autonomic function is less clear although substance P injections into the ventral tegmental area can evoke increases in blood pressure and heart rate (Cornish and van den Buuse, 1995) and neurotensin injections potentiate the pressor response to intravenous vasopressin (van Den Buuse and Catanzariti, 2000). Though unlike the medullary raphe and dlPons, a direct effect on RVLM activity and MSNA by the ventral midbrain remains to be established.

In addition to functional alterations, the dlPons, RVLM, medullary raphe and ventral midbrain displayed increased gray matter volumes in OSA subjects pre-CPAP compared with controls with this altered anatomy returning to baseline levels during CPAP treatment. Although it is not possible in this investigation to determine the precise cellular changes that may have resulted in these increases in gray matter volume, numerous investigations have explored gray matter changes in OSA and found *decreases* in several regions such as in the hippocampus, cingulate, cerebellar, and prefrontal cortices (Macey et al., 2002; Morrell et al., 2003; Canessa et al., 2011). It has been proposed that these volume decreases result from the repeated hypoxic damage that results from extended periods of night-time apneas and hypoxaemia. However, in striking contrast to changes in higher brain centers, we found that within the brainstem, OSA subjects had *increased* gray matter volumes. It is well known that increased use is associated with increases in gray matter volume in relevant brain regions (Amunts et al., 1997; Maguire et al., 2000; Sluming et al., 2002), although we suggest that “over-use” changes are less likely in the brainstem due to its already tonic nature. Furthermore, we found that neither the anatomical and functional changes pre-CPAP nor the recovery of these values during 6 and 12 months of CPAP treatment were correlated to an individual's OSA severity. These results suggest that the anatomical and functional changes that occur within the brainstem of OSA subjects may not be directly related to the severity of OSA but instead may occur to a maximal extent even in individuals with less severe levels of OSA.

Despite the clear role for the raphe and dlPons in autonomic regulation, our functional and structural findings raise an important question: *how do functional and structural changes within the brainstem start?* Although we do not have direct evidence, we speculate that these changes are consistent with a mechanism involving astrocytic activation and modulation of synaptic activity through altered gliotransmission. It has recently been reported that the CIH model of OSA is associated with significant astrocyte activation in cortical areas such as the hippocampus and further this astrocyte activation is not associated with significant neural death (Aviles-Reyes et al., 2010). It is possible that astrocyte activation also occurs in the raphe, dlPons, and ventral midbrain in individuals with OSA which is consistent with increased gray matter volumes. Astrocyte activation can result in a significant alteration of synaptic dynamics (Halassa et al., 2007; Ben Achour and Pascual, 2012) and are involved in brainstem rhythmic activity since astrocytic glutamate toxins reduce inspiratory burst frequency (Hulsmann et al., 2000). It is possible that a similar modulatory effect of glia might also occur in sympathetic regulatory regions and that continuous hypoxia events result in astrocyte activation which in turn results in altered local synaptic dynamics.

### Limitations

There are a number of limitations to this study which require consideration. We did not follow control subjects over a 12 month period to examine reproducibility of the MSNA, fMRI or gray matter concentration measurements and we did not include OSA subjects that did not undergo CPAP treatment. Although with respect to MSNA, others have shown no change in OSA patients who did not receive CPAP when followed up over a year (Narkiewicz et al., 1999b). Very few brain imaging investigations have explored human brainstem structure and function, particularly in a longitudinal manner, however given that in almost all brainstem regions displaying an initial difference between controls and OSA subjects, signal intensity and gray matter concentrations recovered to control levels following 6 months of CPAP treatment and remained relatively stable for the next 6 months, we are confident that the changes reported here are indeed reliable. Further, one must be careful when describing the location of significant clusters in any human brain imaging investigation. Particularly within the brainstem, given the limited spatial resolution of brainstem imaging it is impossible to be completely sure that the significant clusters represent for example the RVLM. However, we contend that they are located in the described regions and given what is known about the roles of such regions in modulating sympathetic drive, it is likely that our descriptions are accurate. We are not able to determine differences in constant neural activity between subject groups but instead we are restricted to assessing MSNA related changes in activity. It might be the case that a particular cortical region is constantly driving brainstem sites such as the RVLM and dlPons which could be assessed in future studies using a technique such as arterial spinal labeling. Finally, although we found no relationship between OSA severity and either fMRI signal intensity or gray matter volume changes, the inclusion of more subjects, particularly with mild OSA, would be required to make this conclusion reliable.

## Author contributions

LH helped collect and analyze data and wrote the manuscript. RF collected data, analyzed data, and helped write the manuscript. LL collected data, analyzed data and helped write the manuscript. DM recruited patients, helped data collection and manuscript writing. VM designed study, collected data, analyzed data, and wrote the manuscript.

### Conflict of interest statement

The authors declare that the research was conducted in the absence of any commercial or financial relationships that could be construed as a potential conflict of interest. The author VM and handling Editor declared a current collaboration (co-hosting a Research Topic) and the handling Editor states that the process nevertheless met the standards of a fair and objective review.
